# The use of Gompertz models in growth analyses, and new Gompertz-model approach: An addition to the Unified-Richards family

**DOI:** 10.1371/journal.pone.0178691

**Published:** 2017-06-05

**Authors:** Kathleen M. C. Tjørve, Even Tjørve

**Affiliations:** Inland Norway University of Applied Sciences, Elverum, Norway; Centrum Wiskunde & Informatica (CWI) & Netherlands Institute for Systems Biology, NETHERLANDS

## Abstract

The Gompertz model is well known and widely used in many aspects of biology. It has been frequently used to describe the growth of animals and plants, as well as the number or volume of bacteria and cancer cells. Numerous parametrisations and re-parametrisations of varying usefulness are found in the literature, whereof the Gompertz-Laird is one of the more commonly used. Here, we review, present, and discuss the many re-parametrisations and some parameterisations of the Gompertz model, which we divide into *T*_*i*_ (type I)- and *W*_0_ (type II)-forms. In the *W*_0_-form a starting-point parameter, meaning birth or hatching value (*W*_0_), replaces the inflection-time parameter (*T*_*i*_). We also propose new “unified” versions (U-versions) of both the traditional *T*_*i*_ -form and a simplified *W*_0_-form. In these, the growth-rate constant represents the relative growth rate instead of merely an unspecified growth coefficient. We also present U-versions where the growth-rate parameters return absolute growth rate (instead of relative). The new U-Gompertz models are special cases of the Unified-Richards (U-Richards) model and thus belong to the Richards family of U-models. As U-models, they have a set of parameters, which are comparable across models in the family, without conversion equations. The improvements are simple, and may seem trivial, but are of great importance to those who study organismal growth, as the two new U-Gompertz forms give easy and fast access to all shape parameters needed for describing most types of growth following the shape of the Gompertz model.

## Introduction

The Gompertz model [1] is one of the most frequently used sigmoid models fitted to growth data and other data, perhaps only second to the logistic model (also called the Verhulst model) [2]. Researchers have fitted the Gompertz model to everything from plant growth, bird growth, fish growth, and growth of other animals, to tumour growth and bacterial growth [3–12], and the literature is enormous. The Gompertz is a special case of the four parameter Richards model, and thus belongs to the Richards family of three-parameter sigmoidal growth models, along with familiar models such as the negative exponential (including the Brody), the logistic, and the von Bertalanffy (or only Bertalanffy) [13][14]. Numerous parametrisation and re-parametrisations of the Gompertz model can be found in the literature, though no systematic review of these and their properties have been attempted.

The purpose of this paper is firstly to review existing re-parameterisations or model forms of the Gompertz model and discuss their usefulness, and secondly to present and discuss revised versions of two useful Gompertz-model forms.

A review of the Gompertz model is useful because of the many re-parameterisations in the literature, and because confusion and lack of accordance have caused diverging traditions or practices, which have developed independently within different fields. Here we review 7 re-parameterisations and a number of versions of one of these.

We subsequently describe two slightly modified or revised Gompertz model forms, which we label the Unified-Gompertz (or U-Gompertz) models, and to our knowledge are mostly new to the literature. We label them “unified” versions, because they together simplify the interpretation of growth rates and the other parameters, in addition to their comparison between models. These possible U-Gompertz forms are again special cases of the Unified-Richards (U-Richards) model as described by Tjørve and Tjørve [14]. As U-models, they have a unified set of parameters, which is comparable across all “U-models”. We will also explain the purpose of choosing one of the two U-Gompertz forms instead of one of the many other versions. Already in an earlier paper [14] we showed how we obtained the logistic model and the von Bertalanffy model from the Richards model by restraining its fourth variable to a given value, thus we can also in this way obtain U-versions of the logistic and von Bertalanffy models from the U-Richards model. However, we failed to discuss U-versions of the Gompertz model. To find these is more difficult, because they are particular limited cases of the Richards model.

We do not present or discuss the linearization of these models, as this is less useful with today’s computers and software. Neither do we discuss differential versions (forms). With modern software, we can study the growth rate across time simply by asking for the first and second derivative of the fitted model, from which one can also graph the change in growth rate across time. Moreover, the models we propose here have easily interpretable parameters that fully characterize the slope. It is also not necessary to obtain the second derivative to discuss the inflection point, also because one of parameters in the models we propose provides us with the time at inflection directly.

Because the Gompertz model and its many re-parameterisations are applied in different fields to different types of growth, the notation differs greatly in the literature. We use a simple common notation in the main equations presented. Still, researchers of growth from different fields should find no difficulty in following the discussions, even though the notation is not that they use in their own work. However, we have sometimes applied some specific notation for fields in the text, where particular re-parameterisations are prominent.

Lastly, we present a genealogy of useful Gompertz re-parameterisations (Appendix 1), as our previous paper [14] only gave the genealogy of the other models in the Richards family, including the negative exponential, the logistic, the von Bertalanffy, and the Richards.

## History

The Gompertz [1] model has been in use as a growth model even longer than its better known relative, the logistic model [2]. The model, referred to at the time as the Gompertz theoretical law of mortality, was first suggested and first applied by Mr. Benjamin Gompertz in 1825 [1]. He fitted it to the relationship between increasing death rate and age, what he referred to as “the average exhaustions of a man’s power to avoid death”, or the “portion of his remaining power to oppose destruction”. The insurance industry quickly started to use his method of projecting death risk. However, Gompertz only presented the probability density function.

It was Makeham [15] who first stated this model in its well-known cumulative form, and thus it became known as the Gompertz-Makeham (or sometimes Makeham-Gompertz) model, a name we encounter for the first time in Greenwood’s [16] discussions. The first attempt to use a least-squares method for the Gompertz model to find the best curve, was attempted, e.g. [17] [18]. However, they did not linearize the model, as is done later, but only log-transformed the values (dependent variable) to make it easier to determine the sum of squares. This method seems to have been used until the 1940s [19], when Hartley [20] proposed to and first explained how to linearize the Gompertz model.

From the 1920s the cumulative Gompertz-Makeham model also rapidly became a favourite in fields other than that of human mortality, for example in forecasting the increase in demand for goods and services, sales of tobacco, growth in railway traffic, and the demand for automobiles [21][22]. Wright [23] was the first to propose the Gompertz model for biological growth, and the first to apply it to biological data was probably Davidson [24] in his study of body-mass growth in cattle. In 1931 Weymoth, McMillin, and Rich [25] reported the Gompertz model to successfully describe the shell-size growth in razor clams, *Siliqua patula*, and Weymouth and Thompson [26] reported the same for the Pacific cockle, *Cardium corbis*. Soon, researchers began to fit the model to their data by regression, and over the years, the common [15] Gompertz model became a favourite regression model for many types of growth of organisms, such as dinosaurs, e.g. [27] [28], birds, e.g. [13] [29] [30] [31], and mammals e.g. [32] [33] including those of marsupials, e.g. [34] [35]. The Gompertz model is also frequently applied to model growth in number or density of microbes [36, 37], growth of tumours [4, 38, 39], and the survival of cancer patients [40].

Several different re-parameterisations of the traditional cumulative Gompertz model are in use. One of the more important was suggested by Zwietering and colleagues [6] for modelling growth in number of bacteria, and is currently one of the most common models in microbial growth [7, 41,42]. Another prominent re-parametrisation of the Gompertz model is the Gompertz-Laird model, proposed by Laird and fitted to tumor growth data [4]. This model is considered especially useful when we want to discuss the initial value (starting point on the x-axis), and it is greatly used also for describing growth in birds and animals, especially poultry [e.g. 9, 43, 44, 45], and livestock [e.g. 46]. However, the model parameters are not easily interpretable without being converted to more useful measurements.

In addition to ordinary monotonically increasing Gompertz re-parameterisations, modellers of microorganisms in food have developed a number of modified monotonically decreasing Gompertz models for (thermal, pressure, or electric field) inactivation kinetics. We will not discuss any of these here, as their interest is limited to this particular type of “growth” studies.

## Notation and model types

**Fig 1:**
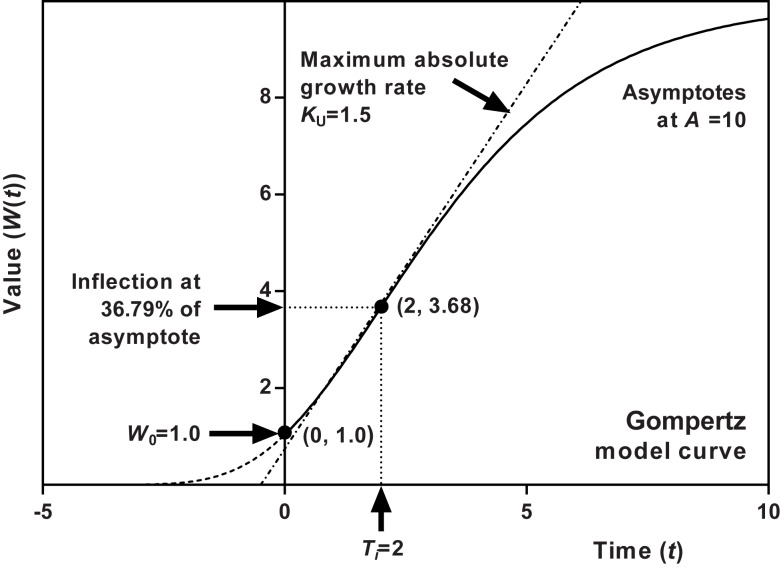
Shape characteristics of the (Unified) Gompertz model (unbroken line). The inflection value is fixed at 36.79% of the upper asymptote. Here the upper asymptote (*A*) is set at 10.0, maximum absolute growth rate (*K*_U_) to 1.5, time at inflection (*T*_*i*_) to 2.0, and the startng point (*W*_0_) to 1.0. With a set asymptote and growth rate, time of inflection follows from a given starting point or vice versa. Maximum growth rate is represented by the tangent at inflection (dashed line).

Here we review Gompertz models found in the literature, focusing on how their parameters affect curve characteristics (Fig 1). We have chosen to present the models using a notation typical for organismal growth studies, describing biometric measurements as functions of time; *W*(*t*). Various fields use different notations, for the value measured, for example survival: *S*(*t*), number of cells/bacteria or population size: *N*(*t*), density of cells or microorganisms; *D*(*t*), concentration of organisms *C*(*t*), volume *V*(*t*), body mass: *M*(*t*), and (f) length: *L*(*t*). The dependent variable (left hand side of the equation) can also be stated as relative values, for example given as *W*(*t*)/*A*, where *A* is the upper asymptote, or *W*(*t*)/*W*_0_, where *W*_0_ is the initial value (or starting point on the x-axis). The latter then represents the value relative to the starting value (described as a “dimensionless” measurement). Sometimes the dependent variable is log-transformed, in particular when modelling microbial growth.

### Two main types of Gompertz models

An important realization is that most Gompertz models can be divided into two groups according to type of location parameter, though this has not yet been called to attention in the literature. Most three-parameter Gompertz models have two “shape” parameters that affect curve shape and one “location” parameter that shifts the curve horizontally without changing its shape. The shape parameters change curve shape but leave the value of the location parameter unaltered. The parameter value is kept constant either relative to the x-axis or relative to the y-axis, characterising type I and type II of Gompertz models, respectively.

In the type I models, a single parameter controls the time (i.e. *x*-value) at which a specific point on the curve occurs. The point represents a fixed proportion (or percentage) of the upper asymptote, and the time at which this point occurs is not affected by the other parameters (though all other points along the curve are). In some models this points falls at the inflection, which in the Gompertz model occurs at 36.8% of the upper asymptote (Fig 1). In other models, it falls at some other fixed percentage of the asymptote.

**Fig 2:**
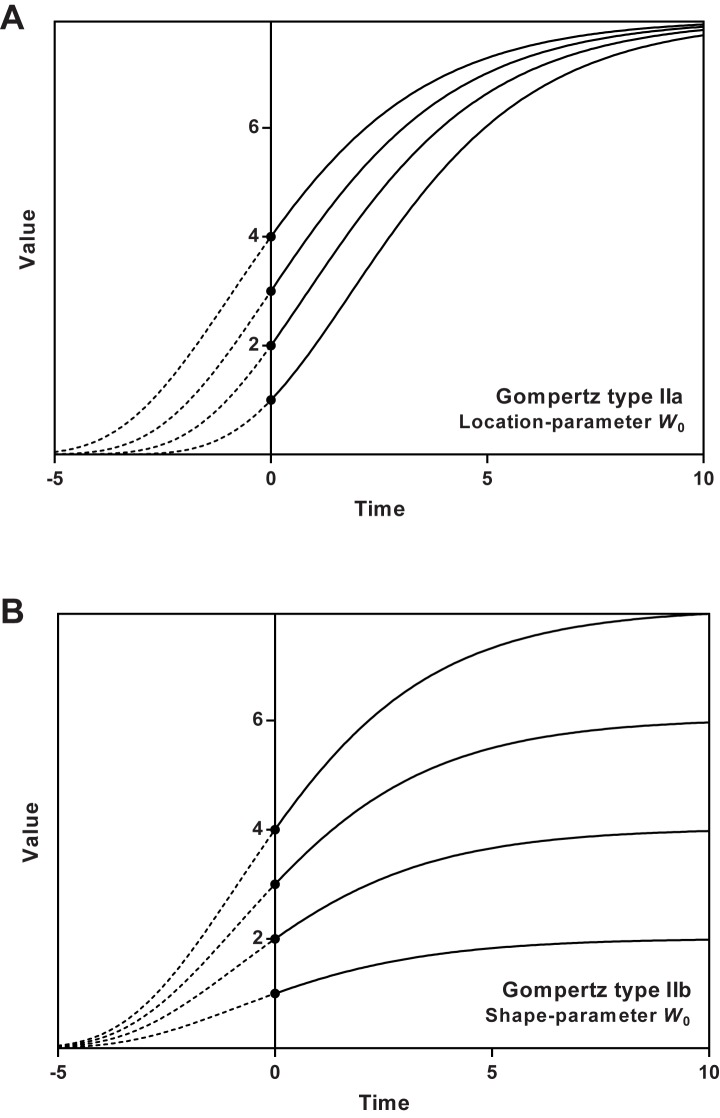
Two kinds of type II models. Both panes show Gompertz curves four different starting-point values (*W*_0_). Panel 2a illustrates how the *W*_0_-parameter affects the curve in type-IIa models (where *W*_0_ acts as a location parameter, keeping the upper asymptote constant), and panel 2b illustrates how the *W*_0_-parameter affects the curve in type-IIb models (where *W*_0_ acts as shape parameter, changing the upper asymptote).

In type II models, a single parameter controls the starting value for the curve (i.e. the intersection with the *y*-axis). In these re-parameterisations, the other parameters do not affect the starting point. Fig 2A and 2B illustrates how the shape parameter changes the curve in a type I model, (Fig 2A) and in a type II model (Fig 2B). Most of the models reviewed here fall into either of these two types, and type II is the commoner of the two.

## Model review

Some of the re-parametrisations of the Gompertz model found in the literature are more useful than others, because they have easy interpretable parameters. One valuable and commonly found re-parameterisation is:

$$W(t)=A\exp\left(-\exp\left(-k_{\text{G}}\left(t-T_{i}\right)\right)\right),$$ (1)

where *W*(*t*) is the expected value (mass or length) as a function of time (for example days since birth or hatching) and *t* is time, *A* represents the upper asymptote (adult value), *k*_G_ is a growth-rate coefficient (which affects the slope), and *T*_*i*_ represents time at inflection. The *T*_*i*_-parameter shifts the growth curve horizontally without changing it shape and is therefore what is often termed a location parameter (whereas *A* and *k*_G_ are shape parameters), which means that this is a type I model. However, more specifically we will refer to model (1) as a *T*_*i*_-form, because *T*_*i*_-is one of the parameters, opposed to the *W*_0_ form (which does not include *T*_*i*_-). We have a *W*_0_-form of a model in the case that *W*_0_ is the value (starting point/intersection) on the y-axis (intersection). All *W*_0_-models are type II. In an earlier work [14] we systemized a number of *T*_*i*_—and *W*_0_ forms for other growth models in the Richards family: the negative exponential, the logistic, and the von Bertalanffy.

Most other re-parameterisations of the Gompertz model found in the literature are less useful, in that their parameters are more difficult to interpret, for example:

$$W(t)=A\exp\left(-\exp\left(-k_{\text{G}}t+b\right)\right),$$ (2)

and

$$W(t)=A\exp\left(-c\exp\left(-k_{\text{G}}t\right)\right),$$ (3)

which are both type II models, but where the *b*-parameter and the *c*-parameter both make the starting point behave as a relative value (a percentage of the upper asymptote), and neither of the two represent the relative value for the starting point (which therefore has been derived from some equation). Thus it is not correct as, for example, Kurnianto and colleagues [47] state, that the *c*- parameter (in model (3)) has “no specific biological significance”. We see that one can convert the location-parameter values between models (1), (2), and (3) from the following equations: *b* = ln(*c*) so that *c* = exp(*b*), *b* = *k*_G_·*T*_*i*_ so that *T*_*i*_ = *b*/*k*_G_, and *c* = exp(*k*_G_*T*_*i*_) so that *T*_*i*_ = ln(*c*)/*k*_G_. Still, we have to conclude that model (1) is more useful than the other two, as we get the *T*_*i*_ parameter directly, instead of having to calculate it.

### The four-parameter Gompertz

In growth-curve analyses of bacterial (or microbial) counts, in particular, the fitting of a four-parameter Gompertz model, as suggested by Gibson et al. [36] [48] (but sometimes erroneously attributed to Jeffries et al. [49]), who only discusses a three-parameter Gompertz), has become commonplace. Using our notation (for comparison), this model becomes:

$$W(t)=B+A\exp\left(-\exp\left(-k_{\text{G}}\left(t-T_{i}\right)\right)\right),$$ (4)

revealing that it is a parameterisation of model (1). The extra parameter in this model, *A*, represents the lower asymptote of the curve, but serves as a location parameter that moves the model curve vertically, without changing its shape. Therefore, the upper asymptote becomes *A*+*B*. The dependent variable, *W*(*t*) (or *L*(*t*), usually described as the log count of bacteria at time *t* [36], meaning that the dependent variable is log-transformed. *A* is typically described as the asymptotic log-count as time decreases infinitely. To be precise, (4) is not a Gompertz model when the dependent variable is log-transformed. However, Jefferies et al. [49] show how model (1) can be log transformed to become:

$$\ln W(t)=\ln A-\exp\left(-k_{\text{G}}\left(t-T_{i}\right)\right).$$ (5)

A simple rewrite of the four-parameter Gompertz, (4), provides what we have referred to as a “compressed form” of the Gompertz model (see [14] for discussion of compressed forms):

$$W(t)=B+(A-B)\exp\left(-\exp\left(-k_{\text{G}}(t-T_{i}\right)\right),$$ (6)

where *B*, which is the lower asymptote, compresses the curve by lifting the lower asymptote without altering the upper asymptote (see also Appendix 1 for a genealogy of Gompertz models).

Both in microbiology (cell or bacteria counts) and in studies of organismal growth, the growth-rate coefficient, *k*_G_, found in many of the Gompertz versions, is often referred to as the “relative growth rate” at inflection (thus maximum relative growth rate). This is incorrect, if we assume that “relative growth rate” must be interpreted as the growth rate given as a proportion (or percentage) of the upper asymptote per time unit, (W/A)·t^–1^. This mistake (or impreciseness) is found both when model (1) [48] and model (3) [36][48] are proposed for microbial growth, and this impreciseness has been copied by a very large number of authors. However, to find maximum relative growth rate (i.e. at inflection and relative to maximum value) we must divide *k*_G_ with the base of the natural logarithm (*k*_G_/*e*). Accordingly, the absolute growth rate is found by multiplying the relative growth rate with the value for the upper asymptote (*k*_G_·A/*e*).

### The Zwietering modification

The re-parameterisation proposed by Zwietering and colleagues [6] is often called a “modified Gompertz” (e.g.[50]) and is typically applied to bacterial growth data, especially in food. It can be given as:

$$W\left(t\right)=A\exp\left(-\exp\left(\frac{e\cdot K_{\text{Z}}}{A}\left(T_{Lag}-t\right)+1\right)\right).$$ (7)

This is a type I Gompertz model where *K*_Z_ is the absolute growth rate (i.e. tangent to the curve) at time *T*_Lag_, termed the “lag time”, which is interpreted as the time between when a microbial population is transferred to a new habitat recovers and when a considerable cell division occurs. *T*_Lag_ falls at where *W*(*t*) = A·exp(-*e*). This means that the so-called lag time (*T*_Lag_) always occurs at the same percentage (6.6%) of the upper asymptote. This means that the location parameter (*T*_*i*_ in models (4) to (6)) is modified to control some other than position than the inflection time.

When this model is fitted to microbial growth data, the dependent variable is typically transformed into the logarithm of the relative population size (ln (*N*(*t*)/*N*_0_, where *N*_0_ is population size at *t* = 0) [6]. An important advantage of the Zwietering re-parameterisation is that the growth coefficient (*K*_Z_) constitutes the absolute growth rate at inflection, and that *A* (the upper asymptote) does not affect this parameter. However, for many types of growth *T*_Lag_ is less intuitive than the *T*_*i*_-parameter.

### The Zweifel and Lasker re-parameterisation

Zweifel and Lasker’s [51] re-parameterisation was copied by Ricker [5] in his book and found its way into the study of fish growth. Today it is often referred to as the Ricker model. It is a good example of a plain type II model where the location parameter represents the absolute value of the starting point on the x-axis. This model is mostly used for fish growth [e.g. 52–57], but it is also fitted to growth data from other animals, for example crustaceans [e.g. 58]. It can be expressed as:

$$W(t)=W_{0}\exp\left(m\left(1-\exp\left(-k_{\text{G}}t\right)\right)\right),$$ (8)

Where the value of the growth coefficient, *k*_G_, is comparable to that in models (1) to (6). *W*_0_ is specified as the initial value (number, density, mass, length etc.). It gives the starting point on the growth curve, though also changing the upper asymptote, because it changes the starting point (intersection with the *y*-axis) by scaling the curve vertically. In addition, the third parameter, *m*, affects the upper asymptote (*A*) by scaling the model vertically. The upper asymptote is found by *A* = *W*_0_ exp(*m*). Both *k*_G_ and *m* affects the time of inflection.

Both Zweifel and Lasker [51] and Ricker [5] used the letter *k* to denote our *m*, some other notation for our *k*_G_, and *t*_0_ to denote our *T*_*i*_ (time of inflection). This has caused some misunderstandings, as the growth coefficient *k*_G_ has erroneously been described as “a dimensionless parameter” and our *m* has erroneously been described as the growth rate. Moreover, *k*_G_·*m* has been described as “growth rate at *t* = 0” e.g. [5]. However, absolute growth rate at t = 0 (the initial growth rate) is *W*_0·_*k*_G_·*m* (and the relative growth rate at *t* = 0 becomes *k*_G_·*m*/ exp(*m*). Moreover, maximum relative growth (found at time of inflection) becomes *e*·*k*_G_·*m*.

This model, (8), is also, as is model (1) (see model (5) above), sometimes encountered log-transformed (e.g.[54] [59]), in our notation stated as:

$$\ln W(t)=\ln W_{0}+m\left(1-\exp\left(-k_{\text{G}}t\right)\right).$$ (9)

### The Gompertz-Laird

Another, and very frequently encountered, type II re-parametrisation is the version of the Gompertz model originally proposed in 1974 by Laird [4][38][60] to describe the growth of tumour size but it is often fitted to growth in numbers (populations) of cells and microbes. The Laird re-parameterisation prevails even today as the most frequently fitted Gompertz version in cancer research, and is now also commonly fitted to growth data in other fields, in particular those of domestic (poultry and livestock) [9, 43–46] and marine (e.g. molluscs, fish, and dolphins) [61–66] animals. It is referred to as the Gompertz-Laird or simply the Gompertz, or even the “modified Gompertz”, as is also model (8). Therefore, one often has to examine the equation to determine whether model (8) or the Gompertz Laird has been used. Moreover, we also found that many authors who stated that they had used the Gompertz-Laird in fact had used some other re-parameterisation, usually model (8).

With the notation of Aggrey [9] (often encountered in studies of growth in domestic animals) the Gompertz-Laird model becomes:

$$W(t)=W_{0}\exp\left(\left(\frac{L}{K}\right)\left(1-\exp\left(-Kt\right)\right)\right).$$ (10)

We may consider this model as a variant of model (8) (or vice versa), but in reality their parameters behave quite differently. The *W*_0_-parameter is comparable to those of model (8), but the other parameters are not. The interpretation of the *K-* and the *L*-parameters vary in the literature and are often ambiguous or not well explained.

The model is again (as is model (10)) unusual in that the *W*_0_-parameter not only changes the intersection with the *x*-axis by repositioning the curve horizontally, but rescales the x-axis, so that all values increase or decrease proportionally. Thereby *W*_0_ (contrary to other models) not only affects the initial value (*W*_0_), but also the upper asymptote, *A*. Thus, while in most of the other (type II) models discussed here *W*_0_ behaves as a location parameter (shifting the whole curve horizontally), in model (10) *W*_0_ turns more into a shape parameter.

The *L*-parameter has been described as “the initial specific growth rate” [9], which is a term that is difficult to understand. In reality, *L* measures neither relative growth nor maximum growth (which falls at inflection). However, the absolute growth rate conveniently becomes *W*_0 ·_*L* at *t* = 0. Thus, *L* is the initial absolute growth rate divided by the initial value. To further complicate interpretation, the *L*-parameter also changes the upper asymptote, *A*, and the inflection time, *T*_*i*_. Consequently, *L* affects three shape characteristics of the growth curve, in addition to growth rate. This makes it difficult to interpret this parameter and compare its values between data sets. It is sometimes erroneously described as maximum relative growth (rate) [67–70].

The *K*–parameter affects both maximum growth and the upper asymptote, as does the *k*_RI_-parameter in model (8). Thus both *L* and *K* affect maximum growth rate (i.e. growth rate at inflection). However, the *K*-parameter affects neither inflection time, nor the initial growth rate (*W*_0_*L*), which are both affected by the *L*-parameter. According to Aggrey [9], the *K*-parameter is the “rate of exponential decay of initial specific growth rate”, a statement copied by many subsequent papers on poultry and livestock growth (see above). This means that it influences how fast the growth curve levels off (towards its asymptote). Thereby *K* also affects the time of inflection, maximum relative growth rate, and upper asymptote.

Summing up which parameters control the three main shape characteristics, we find that *L* and *K* both affect maximum relative growth rate and time of inflection, whereas all three parameters, *L*, *K*, and *W*, together control the upper asymptote. This makes it more difficult to interpret the parameter values of model (10) than for example model (1), where each parameter only affects one of these shape characteristics. The strength of the Gompertz-Laird is the inclusion of the *W*_0_-parameter, which gives us the fitted value at the starting point (and allows us to restrict the starting point by fixing it to a particular value), and that the starting-point growth rate is easy to calculate. The weakness of the Gompertz Laird is the complicated interpretation of its parameters, in addition to the loss of the *T*_*i*_ -parameter (time of inflection) and the *A*-parameter (easily recognizable as time of inflection and asymptote, respectively).

### Simpler W_0_-forms

Another type-2 re-parameterisation, is that suggested by Norton [39]. It is sometimes incorrectly considered to be a Gomperz-Laird model, and is given as:

$$W(t)=W_{0}\exp\left(\ln\left(\frac{A}{W_{0}}\right)\left(1-\exp\left(-k_{\text{G}}t\right)\right)\right).$$ (11)

This model (11) has very different parameters from Laird’s model. It has the same growth-rate coefficient and the same parameter for the initial value (or starting point) as model (8). The model does not (contrary to the Gompertz-Laird) alter its upper asymptote when the starting-point parameter, *W*_0_, is changed. Because the parameters are easily interpretable and control single curve characteristics, Norton’s re-parameterisation is a very useful one. However, we can rearrange this model to a more convenient form:

$$W\left(t\right)=W{\left(\frac{A}{W_{0}}\right)}^{1-\exp\left(-k_{\text{G}}\cdot t\right)}.$$ (12)

This is the exact same model as (11). However, several other and maybe simpler versions of this model can be found, for example the one proposed by Rogers et al. [71], given as:

$$W\left(t\right)=A\exp\left(\ln\left(\frac{W_{0}}{A}\right)\exp\left(-k_{\text{G}}t\right)\right).$$ (13)

An easy way to achieve this model version is to derive it from Eq (3), because *c* = ln (*A*)–ln *(W*_*0*_) (thus substituting ln (*A*/*W*_0_) for *c*, or ln (*W*_0_/*A*) for (–c), which both give model (12)) (see also for example Mignon-Grasteau et al. [72]). (The log-transformed version of (13) is found in Appendix 1). By rearrangement we can rewrite (13) (or indeed also (11) and (12)) into a simpler form (which we have not seen previously in the literature):

$$W\left(t\right)=A{\left(\frac{W_{0}}{A}\right)}^{\exp\left(-k_{\text{G}}\cdot t\right)}.$$ (14)

This re-parameterisation (14) can also be rearranged into other forms (see Appendix 1), which should be recognized as restructured versions of the exact same re-parameterisation of the Gompertz, together Eqs (11), (12) and (13).

Terming model (1) the *T*_*i*_−form and terming model (11) to (14) the *W*_0_-forms of the traditional Gompertz, we designate the simplified model (14) the preferred version. The two complementary models form (1) (the *T*_*i*_-form) and (14) (the *W*_*0*_-form) which supplement each other, because they together provide parameter values for four easily interpretable parameters, each controlling only one shape characteristic. Specifically, *A* controls the upper asymptote; *W*_0_ controls the intersection with the x-axis (starting point), *k*_G_ controls the slope at inflection (maximum growth rate), and *T*_*i*_ controls the age at inflection (age time at maximum growth rate).

Fitting the two model forms to data gives us the exact same curve and values for four parameters, *A*, *k*, *T*_*i*_, and *W*_0_. Therefore, this *W*_0_-form, (14), because it has the *W*_0_-parameter, becomes a useful and simple alternative to fitting the Gompertz-Laird.

### Two kinds of W_0_-parameters

In the above, we described two ways the *W*_0_-parameter may affect the growth curve. In all models, naturally, *W*_0_ controls the starting value (i.e. the intersection with the x-axis). However, by changing its value, one necessarily affects the curve. In some models *W*_0_ acts as a location parameter (Fig 2A) that shifts the curve horizontally without changing its shape. In other models *W*_0_ acts as a shape parameter that scales the whole curve vertically (Fig 2B), thereby affecting the value of the upper asymptote. In other words, the model forces the starting point to behave like a relative value, meaning that when changing its absolute value, it is still locked at a given percentage of the upper asymptote.

This means that we can divide type II Gompertz models into two groups, type IIa (where *W*_0_ is a location parameter) and type IIb (where *W*_0_ is a shape parameter). Model (8) and (10) have *W*_0_-parameters that scale the curve vertically, whereas models (11) to (14) have *W*_0_-parameters that shifts the curve horizontally.

### The Unified-Gompertz

The traditional three-parameter Gompertz model, as the version shown in Eq (1), is a special case of the four-parameter Richards model, for example given as:

W(t)=A(1−(1d)⋅exp(−kR(t−Ti)))d, (15)

where *k*_R_ is the model-specific growth constant controlling maximum growth rate, and the *d*-parameter controlling the inflection value (e.g. mass or length). This model, (15), suffers from the same problem as the traditional Gompertz models, including models (1) and (14), namely that the growth parameter (*k*_G_) is not comparable to growth coefficients in versions of other traditional models, for example versions of the logarithmic model and the von Bertalanffy (which are also species cases of the Richards model). Moreover, these growth parameters (or growth coefficients) are more difficult to interpret because they do not constitute the absolute or relative growth rate. We [14] therefore recommended two Richards-model forms, which we termed the Unified-Richards (or U-Richards). The first of these, the *T*_*i*_-form of the U-Richards [14, 73], is given as:

W(t)=A(1+(d−1)⋅exp(−kU(t−Ti)dd1−d))11−d, (16)

where *d* is the fourth parameter (shifting the inflection value). The second, the *W*_0_-form of the U-Richards, [14] then becomes:

W(t)=A(1+((W0A)1−d−1)⋅exp(−kU⋅tdd1−d))11−d. (17)

Unified versions of the logistic model and the von Bertalanffy model are achieved by substituting the *d*-parameter model (16) and (17) with a constant; *d* = 2 and *d* = 2/3, respectively. However, the Gompertz models are not reached simply by limiting the *d*-parameter to a fixed value, because it is calculated as a limit. This is because these model forms converge to Gompertz models when *d*→1, but *d*≠1 (as the traditional Richards models also do). This means that we achieve U-Gompertz forms by substituting ℯ·*k*_U_ for *k*_G_ in model (1) and (14). The *T*_*i*_-form of the U-Gompertz then becomes:

$$W(t)=A\exp\left(-\exp\left(-e\cdot k_{\text{U}}\left(t-T_{i}\right)\right)\right).$$ (18)

Moreover, the U-Gompertz of the simple *W*_0_-form presented in Eq (14) becomes the natural alternative when we prefer the model to return the starting value (*W*_0_) rather than the inflection time (*T*_*i*_). The *W*_0_-form can then be reformulated to become:

$$W\left(t\right)=A{\left(\frac{A}{W_{0}}\right)}^{\exp\left(-e\cdot k_{\text{U}}\cdot t\right)}.$$ (19)

This also means that by dividing the *k*_G_-value of Gompertz models (1) and (14) with ℯ, we obtain the maximum relative growth rate at inflection, *k*_U_. Thus *k*_U_ = *k*_G_/ℯ = *k*/2.71828. The subscript “U” may notate the universality of this growth coefficient, representing relative growth rate rather than being a mere coefficient.

By re-parameterizing Gompertz forms (1) and (14), the traditional *k*_G_-parameter has given way for the new *k*_U_-parameter in two new model forms, (18) and (19). We term these the *Universal-Gompertz* (or U-Gompertz). In these two forms not only *A*, *W*_0_, and *T*_*i*_ are readily interpretable (as they are in model (1) and (14)), but also the *k*-parameter, *k*_U_, which has become the maximum relative growth rate. The absolute growth rate then becomes *A·k*_U_. Note that *k*_U_ does not affect *T*_*i*_ (as *k*_Z_ does in model (4)), but does affect *W*_0_ (unless *T*_*i*_ = 0, meaning that the inflection point falls at the *x*-axis).

Because the two U-Gompertz forms in essence are the same model, we can also calculate *W*_0_ in model (19) from *T*_*i*_ in model (18) or vice versa, instead of fitting both model forms to the same data set. The conversion equations, replacing *k*_Z_/*A* for *k*_U_, then becomes:

$$W_{0}=A\cdot \exp\left(-\exp\left(e\cdot k_{\text{U}}T_{i}\right)\right),$$ (20)

and

$$T_{i}=\frac{\ln\left(-\ln\left(\frac{W_{0}}{A}\right)\right)}{e\cdot k_{\text{U}}}.$$ (21)

This conversion is possible also between the *W*_0_ and *T*_*i*_-parameters of model (1) and (14). We find these conversion equations by substituting *k*_G_ for ℯ·*k*_U_ in (18) and (19).

In the Gompertz model, the value at inflection (*W*_*i*_) is locked at 36.8% of the upper asymptote, and is calculated as *W*_*i*_ = *A*/ℯ. The *W*_*i*_-value of the U-Richards, however, is controlled by the *d*-parameter, and it is calculated as *W*_*i*_ = *A*/*d*^1/(1–*d*)^_._ It is an important feature that the *k*_U_ -parameter (maximum relative growth rate) in the U-Richards model, (16) and (17), is the same as in new U-Gompertz model, (18) and (19). This means that if both models are fitted, either to the same or to different data sets, the *k*_U_-parameter can be compared between the two models without any conversion equation. This also holds for the other models (U-logistic and U-Bertalanffy) in the U-Richards family [14]. The growth constants of the traditional models (logistic, Gompertz, von Bertalanffy, and Richards) are, unfortunately, not directly comparable.

#### Absolute growth rate

Earlier authors have also noted, more or less explicitly, that it is possible to re-parameterize the Gompertz model so that the growth parameter returns a relative or an absolute growth rate, as in model (7), above; although the growth rate in this model [6] is the absolute rate at the starting point (*t* = 0) rather than at time of inflection (*T*_*i*_) (e.g. model (18) and (19). However, we may re-parameterise models (18) and (19) in order to return absolute instead of relative growth rates at inflection (*K*_U_), i.e. maximum absolute growth rates. The *T*_*i*_-form of the U-Gompertz model (18) then becomes:

$$W(t)=A\exp\left(-\exp\left(-\frac{e\cdot K_{\text{U}}\left(t-T_{i}\right)}{A}\right)\right),$$ (22)

and the W_0_-form of the U-Gompertz (19) becomes:

W(t)=A(AW0)exp(−e⋅KU⋅tA). (23)

This offers a choice between *W*_0_-type models that return maximum absolute growth instead of relative growth. Whether one chooses model (18), (19), (22), or (23) depends on which parameter value is most convenient to discuss and to compare between data sets. If the purpose is to compare statistically values between data sets, one should fit the model that returns the value; absolute or relative growth rate, that one wants to compare. The software then usually provides standard errors (or confidence intervals) for the parameter values

## Conclusion

This article’s main contributions are the new U-Gompertz model forms, and pertaining deliberations. The changes relative to traditional models are simple, and may seem trivial, but are of great importance to those of us who study organismal growth. The two new U-Gompertz forms provide easy interpretation of all shape parameters, also because each parameter only affects one shape characteristic. In addition, the parameter for maximum growth rate is comparable to the growth-rate parameter of all models in the U-family. This makes it easier to describe most types of organismal growth following the shape of the Gompertz model and to compare fitted parameter values across models. Moreover, confidence intervals are easily calculated for estimated parameters, but are difficult to obtain from derived measurements that have to be calculated from parameter values. Therefore, directly biologically interpretable parameters are preferable, like those returned by the two U-Gompertz forms. Being able to calculate confidence intervals for the fitted values, we can also compare these values between data sets, by applying for example a t-test or an ANOVA.

This development also fills a gap in our 2010 paper [14], which reviews and discusses the U-model family, including re-parametrisations of the negative exponential, logistic, and von Bertalanffy models, but not the Gompertz model. In addition, we present a rearrangement, which we have not seen in the literature, of the *W*_0_-version of the traditional Gompertz model in a simpler form.

When studying growth, one is sometimes more interested in the starting point, *W*_0_, of the curve than the exact upper asymptote, as should be the case in growth studies of poultry and livestock more than in wild birds and mammals. Then one will probably want to choose a model that directly returns a *W*_0_-value. The *W*_0_-form of the Gompertz, and preferably the U-Gompertz, is a good alternative to the Gompertz-Laird. The *L*-parameter of the Gompertz-Laird (which does not have a simple interpretation) and the lack of an *A*-parameter are problematic in this model. We believe that both the *T*_*i*_ and the new *W*_0_-form of the U-Gompertz model gives easy and fast access to the shape parameters needed for most types of growth studies. Because of its *W*_0_-parameter, the *W*_0_-form of the U-Gompetrz promises to be a useful alternative also to the traditional Gompertz-Laird. The U-Gompertz forms are even alternatives to the two U-Richards forms, when a three-parameter model is preferred. Still, the usefulness of the U-models, like the U-Richards and the U-Gompertz, and their *W*_0_-forms in particular, have yet to be firmly established, though the *W*_0_-form of the U-Richards has already been successfully fitted to, for example, the growth of yoghurt bacteria [74][75], and the growth of wader chicks [76][77][78].

## Supporting information

S1 Appendix A geneaology of some useful and reccomended Gompetz models and their U-model versions. (DOCX)
