# Supplementary material for: The use of Gompertz models in growth analyses, and new Gompertz-model approach: An addition to the Unified-Richards family
Source: PLoS One. 2017 Jun 5;12(6):e0178691. doi: 10.1371/journal.pone.0178691 (PMC5459448; doi:10.1371/journal.pone.0178691)
Supplement: S1 Appendix — (DOCX) [file pone.0178691.s001.docx]

**Appendix:** A geneaology of some useful and reccomended Gompetz models and their U-model versions. The main difference between the ordinary Gompertz models and their U-Gompertz counterparts is that the traditional growth coefficient (*k*_G_) is replaced by a parameter that returns the actural relative growth rate at inflection. Here we show U-versions of both growth-rate parameters that return relative maximum growth rates (*k*_U_) and absolute maximum growth rates (*K*_U_). We state that *k*_G_ = *e*· *k*_U_ = *e*·*K*_U_ /*A*.

|  | **Gompertz with traditional growth coefficient** (*k*_G_) | **Gompertz U-models returning relative maximum growth rates** (*k*_U_) | **Gompertz U-models returning absolute maximum growth rates** (*K*_U_) |
| --- | --- | --- | --- |
| ***T_i_* –form**  **(type I)** | $A\exp\left(-\exp\left(-k_{\text{G}}\left(t-T_{i}\right)\right)\right)$ | $A\exp\left(-\exp\left(-e\cdot k_{\text{U}}\left(t-T_{i}\right)\right)\right)$ | $A\exp\left(-\exp\left(-\frac{e\cdot K_{\text{U}}}{A}\left(t-T_{i}\right)\right)\right)$ |
| **ln of *T_i_* –form**  **(type I)** | $\ln A-\exp\left(-k_{\text{G}}\left(t-T_{i}\right)\right)$ | $\ln A-\exp\left(-e\cdot k_{\text{U}}\left(t-T_{i}\right)\right)$ | $\ln A-\exp\left(-\frac{e\cdot K_{\text{U}}}{A}\left(t-T_{i}\right)\right)$ |
| ***W*_0_ –form**  **(type IIa)** | $W_{0}\exp\left(\ln\left(\frac{A}{W_{0}}\right)\left(1-\exp\left(-k_{\text{G}}t\right)\right)\right)$ $A\exp\left(\ln\left(\frac{W_{0}}{A}\right)\exp\left(-k_{\text{G}}t\right)\right)$ $W\left(\frac{A}{W_{0}}\right)^{1-\exp\left(-k_{\text{G}}\cdot t\right)}$ $A\left(\frac{A}{W_{0}}\right)^{-\exp\left(-k_{\text{G}}\cdot t\right)}$ $W_{0}\left(\frac{W_{0}}{A}\right)^{\exp\left(-k_{\text{G}}\cdot t\right)-1}$         | $W_{0}\exp\left(\ln\left(\frac{A}{W_{0}}\right)\left(1-\exp\left(-e\cdot k_{\text{U}}t\right)\right)\right)$ $A\exp\left(\ln\left(\frac{W_{0}}{A}\right)\exp\left(-e\cdot k_{\text{U}}t\right)\right)$ $W\left(\frac{A}{W_{0}}\right)^{1-\exp\left(-e\cdot k_{\text{U}}\cdot t\right)}$ $A\left(\frac{A}{W_{0}}\right)^{\exp\left(-e\cdot k_{\text{U}}\cdot t\right)}$ $W_{0}\left(\frac{W_{0}}{A}\right)^{\exp\left(-e\cdot k_{\text{U}}\cdot t\right)-1}$         | $W_{0}\exp\left(\ln\left(\frac{A}{W_{0}}\right)\left(1-\exp\left(-\frac{e\cdot K_{\text{U}}t}{A}\right)\right)\right)$ $A\exp\left(\ln\left(\frac{W_{0}}{A}\right)\exp\left(-\frac{e\cdot K_{\text{U}}t}{A}\right)\right)$ $W\left(\frac{A}{W_{0}}\right)^{1-\exp\left(-e\cdot K_{\text{U}}\cdot t/A\right)}$ $A\left(\frac{A}{W_{0}}\right)^{\exp\left(-e\cdot K_{\text{U}}\cdot t/A\right)}$ $W_{0}\left(\frac{W_{0}}{A}\right)^{\exp\left(-e\cdot K_{\text{U}}\cdot t/A\right)-1}$         |
| **ln of *W*_0_ –form**  **(type IIa)** | $\ln W_{0}+\ln\left(\frac{A}{W_{0}}\right)\left(1-\exp\left(-k_{\text{G}}\cdot t\right)\right)$ $\ln W_{0}+\ln\left(\frac{W_{0}}{A}\right)\exp\left(-k_{\text{G}}\cdot t\right)$   | $\ln W_{0}+\ln\left(\frac{A}{W_{0}}\right)\left(1-\exp\left(-e\cdot k_{\text{U}}\cdot t\right)\right)$ $\ln W_{0}+\ln\left(\frac{W_{0}}{A}\right)\exp\left(-e\cdot k_{\text{G}}\cdot t\right)$   | $\ln W_{0}+\ln\left(\frac{A}{W_{0}}\right)\left(1-\exp\left(-\frac{e\cdot K_{\text{U}}\cdot t}{A}\right)\right)$ $\ln W_{0}+\ln\left(\frac{W_{0}}{A}\right)\exp\left(\frac{e\cdot K_{\text{U}}\cdot t}{A}\right)$   |
| **Compressed *T_i_* –form (type I)** | $B+(A-B)\exp\left(-\exp\left(-k_{\text{G}}(t-T_{i}\right)\right)$ | $B+(A-B)\exp\left(-\exp\left(-e\cdot k_{\text{U}}(t-T_{i}\right)\right)$ | $B+(A-B)\exp\left(-\exp\left(-\frac{e\cdot K_{\text{U}}}{A}\left(t-T_{i}\right)\right)\right)$ |
